# Retracted: PPARs Expression in Adult Mouse Neural Stem Cells: Modulation of PPARs during Astroglial Differentiaton of NSC

**DOI:** 10.1155/2019/5656198

**Published:** 2019-04-30

**Authors:** PPAR Research

*PPAR Research *has retracted the article titled “PPARs Expression in Adult Mouse Neural Stem Cells: Modulation of PPARs during Astroglial Differentiaton of NSC” [1]. The article was found to include image duplication and splicing. The details, as raised on PubPeer and confirmed by the Editorial Board, are as follows:

(1) Figure 4 RXR alpha is the same as Figure 4 RXR gamma. Figure 7(d) RXR gamma is the same, but stretched horizontally.

(2) Figure 4 RXR beta may have a splice line at the right-hand side of the band.

(3) In Figure 8, the marker lanes and actin band appear to be the same in all three panels. Each panel shows at least three rectangular blocks separated by splice lines when the contrast and exposure are adjusted. The top panel appears similar to the middle panel when the contrast and exposure are adjusted.

Additionally, the article was found to contain a substantial amount of material from the article cited as reference [14], where 684 words overlap:

Jérôme N. Feige, Laurent Gelman, Liliane Michalik, Béatrice Desvergne, Walter Wahli. "From molecular action to physiological outputs: Peroxisome proliferator-activated receptors are nuclear receptors at the crossroads of key cellular functions", Progress in Lipid Research, 2006. https://doi.org/10.1016/j.plipres.2005.12.002  [2].

The text overlap is mainly found in the Abstract and the Introduction.

We asked the authors for their explanation and to provide the uncropped and unadjusted images underlying all the figure panels. The first author explained that the Western Blots in Figures 4 and 7 are the same because this is the same result presented in both figures, i.e. no expression of RXR gamma, the initial conditions for both undifferentiated and differentiated cells. The actin bands in Figure 8 were cut and included with the corresponding bands for each PPAR isotype to better present the results. Actin and each PPAR isotype were run in separate wells and amplified with different PCR conditions. However, they could not provide the original files because of a 2009 earthquake.

The editorial board agreed with retraction, but the first author did not.
